# London without Hospitals

**Published:** 1891-12-19

**Authors:** 


### The Hospital

A Weekly Journal of

Science, Medicine, Nursing, and Philanthropy.

*For Hospitals, Asylums, Infirmaries, Dispensaries, Medical Practitioners, Students, Nurses, and the Charitable Public.*

Vol. XI.—No. 273.] [Dec. 19, 1891.

### London Without Hospitals.

That this is not the best of all possible worlds is candidly admitted, but it is the best of which any of us have any personal experience, and it is clearly our interest to make the best use we can of it.

When the present writer was selected by the suffrages of his companions in arms to prepare the chief dish for The Hospital Christmas Number, he felt himself, to use a flippant expression, "knocked into a cocked hat." "London Without Hospitals!" The theme is overpowering! Hogarth painted London without morals; but where is the Hogarth who shall picture London without Hospitals?

In every piece of work a man must begin somewhere: the question now is, where? If Edgar Allen Poe had had to write of London without hospitals he would probably have begun in some horrible pestilence den, and carted off the struggling living with the still warm dead to be buried in a common grave. If Hogarth had been asked to illustrate the subject with his pencil, he would have depicted "Gin Lane," or a new "Progress of Cruelty," with drunken waggoners driving their lumbering teams over prostrate children, or crowds of leprous beggars displaying their horrid sores on the pavement to the gaze of the passing crowd. All this would have been effective and true. But changed times have brought changed manners. Hogarth disgusts people of modern tastes, all, at any rate, but the critical few; and Edgar Poe has no more charms for the thousands of the present generation than has the croaking of the midnight raven, of which he was the wizard and the poet.

### Superior Persons.

The deeper a man searches into a subject the more he learns about it, and the more clearly he becomes aware of its faults, if it have any. No honest enquirer, investigating the past and present history of hospitals, will feel inclined to indulge in the language of indiscriminate laudation. They have had, and they still have faults. If they could be carried on without the aid of men and women they might conceivably be faultless. But wherever the human creature comes upon the scene, there comes infirmity. It cannot be helped, can it? Many writers make no allowance for human infirmity; they are so thoroughly possessed by that most unpleasant of all infirmities—self-admiration—that they can see no perfection in any of their neighbours, and nothing but perfection in themselves. Such people are not pleasant guests at Christmas time. They quarrel with the goose because it is too fat, and with the turkey because it is too lean. They spoil the flavour of the pudding by their atrabilious tongues, and turn the port sour by their disdainful sippings. They are altogether too superior for common-place mortals. They ought to have a world to themselves, and for our part we shall cheerfully bid them "God-speed" when they set out in search of it.

For ordinary men ordinary things will do, though we are all pleased when our own fortune endows us with what is choicest and best. Are hospitals ordinary, or are they above what is ordinary? Do they belong to the category of good things, or are they properly placed among those that are better than the best? If there are any "best" among the generous institutions of England, hospitals have a first place in the list.

### Norman William and the Peasants.

It is not, after all, so very difficult to imagine in a general kind of way what London would be without hospitals. The difficulty is to bring the imagined picture within limits, and to make the various details so definite that persons of no imagination shall be enabled to realise the situation as clearly as those whose imagination is vivid. When Norman William, the tanner's grandson, reigned over England, some of his more northerly subjects objected to a foreign king, and made a rising, with the patriotic object of driving him over the seas. William swooped down upon them with his trained warriors; and they, being for the most part peasants led by their feudal lords, were quickly put to the rout. The Norman, by way of convincing the whole island that patriotism of that kind did not pay, burnt the villages and homesteads, destroyed the crops, and killed as many of the inhabitants as he could over a range of sixty miles of country.

There were in those days no field ambulances, no hospitals, no surgeons, no nurses. Happy were the peasants who received wounds that proved quickly mortal in the fight! Thousands of those who were less severely injured, and thousands more of their wives and little ones, would drag themselves into the woods and remoter regions of the hills, there to die by inches; whilst the fox, the stoat, and the wild cat by night, and the eagle, the hawk, and the carrion crow by day, would feed upon the dead, and often perhaps upon those who were still living and conscious, but had not the power to drive the ravenous beasts away.

William made a desolation, and called it "peace." But if every reader would imagine himself a wounded peasant, dragging out days of slowly-ebbing life on the cold hill side, whilst eyes hungry for carrion watched day and night until his nerveless limbs should announce that the feast was ready, how devoutly he would thank Providence that on every civilised battle-field there are ambulances ready, with skilled surgeons, kind and gentle nurses, and a protecting hospital in the rear. Now, the brave soldier is not left to a slow and agonising death; but his wounds are bound up, and his aching limbs laid to rest; his fever is assuaged, and his sinking life restored to hope and health. What soldier would choose to fight in the tenth century who could fight in the nineteenth?

### More Horrible than Hogarth.

There was a London of comparatively recent history which was much more horrible than anything that Hogarth ever painted. Hecker, in his *Epidemics of the Middle Ages*, gives an account of the "Black Death" and its consequences which visited London and all other English and European towns and cities in the fourteenth century. The peculiar symptoms and characteristics of that dreadful malady were almost too terrible for reproduction. The victims were stricken by an ardent fever. In many cases blood gushed almost simultaneously from the nose, the mouth, the urinary passages, and the bowels. Huge swellings appeared on the legs and arms, in the groins, and in the armpits. In many cases black spots broke out all over the body; spots which sometimes remained separate, but not seldom ran together, converting the whole skin into a foul covering of putridity. Many of the patients became stupefied, with their tongues paralysed, and fell into a profound slumber which proved the sleep of death. Others, less happy, could not sleep at all, but were tossed with restless agonies, and parched with burning thirst until death relieved them. The plague was so contagious that almost all persons who approached the sufferers were immediately smitten, and died within a few days. Boccaccio averred that he saw two hogs tearing about the rags of a person who had died of the plague. Almost immediately the animals began staggering about as if they had taken poison, and speedily fell down dead. Multitudes of dogs, cats, fowls, and other domesticated creatures were everywhere infected, and in their madness carried the horrible malady in all directions. In London alone fifty thousand persons, one in every ten of the whole population, are recorded to have died.

### No Hospitals, No Help, Civilisation Collapsed.

There were no hospitals in those days, no buildings in which the plague-stricken could be separated from the healthy, no floating vessels on board which they might have the benefits of uncontaminated air; above all, no scientific physicians who understood the nature of the terrible problems to be solved, and no trained nurses; in short, no effective means of any kind for dealing with so ruthless and devastating an enemy. Physicians, priests, and people alike were paralysed by the overwhelming flood of pestilence. Those who felt themselves attacked fell down in intolerable anguish and despair where they were seized. We can picture the terrible scenes: the narrow and undrained streets and roadways choked with plague-stricken forms: the wretched houses with their filthy rooms filled with the curses of the living, the groans of the dying, and a horrible stench from the dead, which carried the fatal poison into the veins of all who inhaled it. But no words can approach a realistic description of the horrors of such a time. Yet it is but five hundred years since all this happened.

It is often taken for granted, even by intelligent persons, that a fatal epidemic bears down like a flood upon a devoted population, and passes away, also like a flood, in a few days or weeks, leaving no traces behind. But the very opposite is the actual truth. The Black Death visited England in 1342, and remained to scourge and terrify the inhabitants for six years, until 1348. Even that was only a small part of the horrors which followed in its train. The general effect of that terrible visitation can only be described as a collapse of civilisation for a period. Hecker, indeed, affirms that the consequences have continued to be felt for centuries; that a "false impulse" was then communicated to civil life, which in England has extended even to modern times.

### Religion Paralysed, Morals Destroyed, Business Ruined.

One might suppose that such a visitation would be eminently favourable in its after consequences to religion and public and private morals. The truth, however, was that for a long time morals and religion were utterly paralysed and destroyed. Over large tracts of country the churches were entirely deserted and public worship was no longer carried out. The schools shared the fate of the churches, and education ceased to be desired. Ignorance, grossness, barbarity, and animal selfishness everywhere prevailed. With a touch of grim humour Hecker tells us that one class alone prospered in the midst of the general degradation. Those were the lawyers of the period. "Covetousness," says the chronicler, "became general; and when tranquility was restored the great increase of lawyers was astonishing, to whom the endless disputes regarding inheritances offered a rich harvest. The sittings of Parliament, of the King's Bench, and of most of the other courts were suspended as long as the malady raged. The laws of peace availed not during the dominion of death."

The practical business of life, as well as its religion and its law, was for a time almost entirely suspended. The amenities of civilization were forgotten, and the restraints of public opinion ceased to operate. The lowest and worse passions of men came uppermost, and were indulged without let or hindrance. England became practically a savage country for several years. The fields were in many places untilled, and the cattle, for want of herdsmen, ran wild in the forests and on the hills by tens of thousands. The whole country was thrown backwards in its development by a period of years which cannot be computed. "At the commencement of the epidemic," says the historian, "there was in England a superabundance of all the necessaries of life; but the plague, which seemed then to be the sole disease, was soon accompanied by a fatal murrain among the cattle. Wandering about without herdsmen, they fell by thousands; and the birds and beasts of prey are said not to have touched them. In consequence of the murrain and the impossibility of removing the corn from the fields, there was everywhere a great rise in the price of food." The wholesale destruction of the cattle and the crops, following so closely upon the devastation and death which everywhere accompanied the epidemic completed a picture of desolation and ruin of which adequate description is impossible.

It is necessary to recollect, what has already been stated, that all this happened little more than five centuries ago. Compared with many of the prominent facts of history, the Black Death was quite a modern event. The writer does not hesitate to say that such a visitation ought to have been impossible. It could not have happened except in the complete absence of hospitals, nurses, and scientific medical resources. It was a disgrace to the civilization and science of the times. Nothing more convincingly shows the poor mental capacity of the average man than the miserably ineffective way in which the approach of the epidemic was met. The physicians capitulated at the first appearance of the enemy. The priests were powerless, and the public authorities might as well have been non-existent. The plague took absolutely its own course, exactly as it would have done in the Britain of the Druidical period, or in the most savage regions of the Darkest Africa of to-day.

### Then and Now.

But if we were to be threatened, or even attacked by such a devastating foe at the present time, what a contrast there would be between the way in which our forefathers met the enemy and the way in which we should meet it. We are—that is, the medical profession and the hospitals are—armed at every point, and thoroughly equipped like a splendid army. Germany thinks it necessary to arm and drill its population in order to be always ready to resist the attacks of an invading foe. But large masses of people, gathered together in great towns, and with inadequate sanitary arrangements, are in much more constant danger from unseen foes that crowd the drains, the waters, and the surfaces of polluted rivers, the dark mists of marshy districts, and even the very atmosphere of thickly populated neighbourhoods, than Germany is from a foreign foe. Those unseen destroyers are always in a condition of perfect drill, always full armed for the fight, always ready when some directing circumstance points the way, to swoop down upon ten men or ten thousand, and to annihilate them with a single charge of their invisible battalions.

The man who has a wide acquaintance with the history and the possibilities of bacterial warfare often stands appalled in the midst of our civilization. Think, for example, of the thousands of miles of underground drainage, great and small, over which London stands as over the mouth of a pestilential and deadly inferno! Every mile of that drainage is charged with poison sufficient to kill hundreds of the strongest men. Britain is but a small island; and yet she has a population equivalent to that of seven Londons, all living upon the arched roofs of seven similar infernos. These facts make the scientific imagination stand aghast in almost mortal terror. But we must think also of the deadly fevers that are always either smouldering or raging in our midst. Where is there a town of any magnitude that has not always within its area some cases of scarlet fever, diphtheria, typhoid, small-pox, or other disease of poisonous bacillary origin? Each of these active fever cases is a central fire, limited for the moment, it is true, to a particular point, but surrounded on all sides by myriads of deadly bacilli that constitute the inflammable gunpowder, upon which every case of zymotic fever may at any moment act as a lighted match, and spread conflagration and destruction on all sides.

But, thanks to hospitals and scientific medicine, and to them exclusively, we are able to deal with every outbreak of zymotic disease on its first appearance, and to deal with it intelligently, scientifically, and effectively. We know what we are doing; we know the character of the foes we have to deal with; we understand thoroughly what they can do, and what they can not do. Above all, we know how to circumvent them, to defeat them, and to crush and destroy them. Even in those rare cases where they do make considerable headway, we can catch them up, check them, and mitigate their direful consequences. We do not stand in their presence paralysed and helpless, and give ourselves up to despair. We have the boldness and the authority of knowledge, and we enter upon the conflict against our dangerous foes with hearts animated by intelligent hope, and the confidence of assured victory.

### Every-day Sufferings.

Thus far, we have dealt with the exceptional circumstances of war and pestilence. But if those circumstances were found to demand hospitals, with skilled physicians and surgeons, and kindly nurses, how much more do the common and every-day sufferings and dangers of the sick poor plead with irresistible urgency for the consideration and care of the well-disposed? Hunger does not the less demand to be appeased because it recurs every day than it would do if it were experienced once a month, or once in twenty years. It is still hunger; and, as Carlyle says, "The imperative nature of hunger is well known." So also the common diseases, from which men are suffering all the year round, inflict as much agony or kill with as infallible a certainty as does pestilence, or famine, or war. Let us suppose that all of a sudden every hospital in London is wrecked by an earthquake, and all the doctors and nurses are swallowed up at the same moment! What shall we do with our sick, who are numbered by tens of thousands, who have no money, and whose friends have no money?

### A Dead-lock in the City.

A deadlock like what is here supposed once actually happened in our own country. When Henry VIII. dissolved the religious houses there were three institutions in London to which the name of hospital, as we understand it, could with some propriety be applied. Those were St. Bartholomew's, St. Thomas's, and Bethlehem. They all shared the fate of the religious establishments, and were compulsorily closed. What was the result? The whole of their inmates were turned into the street. But many of them had neither home, friends, nor money. The street was their only refuge, and the tender mercies of the half savage crowd their only help. Such a state of things was speedily found to be intolerable, and that not by the priests and gentle-hearted religious women of the period, but by money-loving, practical, and worldly-minded men. It was the business men of the period, the bankers, the merchants, and the manufacturers who found themselves compelled to take the matter in hand. The Mayor, with the Aldermen and the Common Council, drew up and presented a petition to the king in the thirteenth year of his reign, for the reopening of the hospitals which had been closed, and for the restoration of all their resources for the care and cure of the sick poor.

The petition of the Mayor and Aldermen sets forth, in the language of the times, that the object of their prayer was "The ayde and comforte of the poore sykke, blynde, aged, and impotent persones beyinge not hable to helpe themselffes, not havynge any place certeyn wheryn they may be lodged, cherrysshed, and refresshed tyll they be cured and holpen of their dyseases and syknesse." The prayer goes on to describe "the myserable people lyenge in the streetes, offending every clene person passyng by the way with their fylthye and nasty savours," and ends by beseeching the king "that the *Mayor and his Brethren* of your Cytye of London, or with such other as shall stande wyth your most gratyous favour, shall and may from henceforthe have the order, rule, dyspocion, and govournance of the sayd hospytalls with the rents appertaynyng to the same, so that the sick, needy, and indygent persons shall be refreshed, mayntayned, and comforted, fownde, heled and cured of theyre infyrmyties frankly and freely, by phisycions, surgeons, and appotecarys, which shall have *salary*, *stypend*, and *wages* only to attend for that intent and purpose.

The Mayor and Corporation of the London of that period did exactly what might have been expected of them. The Lord Mayor and Corporation of the London of to-day would act in a precisely similar way if they were placed in similar circumstances. The truth is, that the English people have acted according to their feelings in making such noble provision for the care and cure of the sick. They could not have acted, and they could not now act otherwise. Their feelings would get the better of them, and insist upon having their way.

### The Critic Converted.

A great many persons who have never seen the inside of a hospital, and who have had no experience whatever either of sickness or of poverty, in their own persons, look upon hospitals from an outside and purely abstract standpoint, and criticise them with a great deal of freedom and much apparent hostility. What would those critics do if they were suddenly brought face to face with all the sick poor lying helpless and filthy in the street, as Henry the Eighth's Mayor and Aldermen were? Before answering this question, let us consider what the actual sight would be if all the in-patients of the London hospitals were suddenly turned into the street? If the pavement on one side of the road were given up to the beds of those poor persons, we should require a street twenty-five miles long to hold them all; eight thoroughfares, stretching from the Marble Arch to the Bank, would be filled by them. The twenty thousand beds, occupied by persons suffering from every conceivable disease of a painful, loathsome, or dangerous nature, would present a spectacle unparalleled in the world's history. What is the adverse critic going to do with them all? Before he has traversed one quarter of the twenty-five miles of sick-occupied street, he will be utterly and for ever ashamed of his own ignorance and enmity. He will say: "Only take away these people from the cold inclemency of the weather, or from their impoverished and filthy homes; put them into a clean and wholesome shelter; give them the food, the medicine, and the kind care they need for their restoration to wholesomeness and health, and I, the quondam critic will devote a full tithe of my income to the good work of their comfort and maintenance all the days of my life."

That is the way to look at the thing. The ten thousand sick poor in the voluntary hospitals of London, and the other ten thousand in the poor law infirmaries, are an actual and concrete reality. They may be seen and handled every day by anybody who wishes to intelligently realise the situation. Moreover, most of them, at any rate of the ten thousand in the voluntary hospitals, are well-doing, self-respecting, honest, and industrious citizens. Misfortune is their only fault. They have been overtaken by sudden calamity. "Leave them to starve and die"—will any critic say? No! No critic will say that! Then what, in the name of logic and common sense, is the only reasonable alternative? Care for them, cure them with all possible promptitude and skill, and send them back to their work again, when they will once more provide for themselves and their belongings. Is not that the only thing to be done? You notice a fault here and there in the management of hospitals? True! But what business man would bar the Thames out of London because he saw a small shark in the water? The Thames, even with all its horrible foulness, is indispensable to our very existence. We never quarrel with it, or try to diminish its magnitude by a single drop. Even so is it with our hospitals! They are indispensable; a part of our resources, our national character, ourselves! We cannot diminish their effectiveness by the suppression of a single bed. Improve them by all means, when they need improvement, exactly as you would improve the Thames. But, even whilst you are improving them, support them; and always better and better. The very best of all the methods of improving hospitals is to support them by money with the one hand, and by personal interest and watchful attention with the other. There is no help which is so serviceable to the London hospitals as that of an outside critic who has been converted into a life Governor. The writer himself began as a critic, and ended as a life Governor. He has studied hospitals both from the outside and the inside; and he is an enthusiastic convert to the hospital faith.

### Whittington or Our Own Lord Mayor?

Our own generation and the doings of our own times ought not to be less interesting to us than the doings of those who lived five hundred years ago where we live now. Much as we esteem Hogarth's industrious apprentice, and Mr. Richard Whittington of illustrious memory, an actual and living Lord Mayor is more pertinent for present-day purposes. On the ninth of November just past the writer was suddenly hailed by a friend and informed that there were some men bringing an accident case to his consulting room. He hurried to the door, and met the procession approaching. He suggested that as the accident was a severe one, and St. Bartholomew's was but a little way off, the girl, for it was a girl, had better be carried there. Almost before the words were out of his mouth a couple of policemen marched promptly up, pushing in front of them one of the two-wheeled ambulances with which London has been furnished by the Hospitals Association. The girl was gently lifted on to the hammock-like stretcher on wheels, and taken along the asphalte of Cheapside without so much as feeling a single jolt or jar all the way to the hospital. Was it not worth while to have such an ambulance, and such a hospital, for such an emergency?

### Prince George of Wales.

His Royal Highness Prince George of Wales has just passed through the stages of a moderately severe attack of typhoid fever. The illness has extended over five or six weeks, and the convalescence will not be completed in less than five or six weeks more. Now typhoid fever is common in London, and will remain common so long as the water we drink, and the drains that traverse the streets beneath our feet are such as they are. Working men are peculiarly subject to typhoid fever, because the filtering and boiling of drinking water and milk are not properly carried out in their homes, and also because the drainage and general sanitation of their houses are for the most part bad. Think now of one aspect of the case of a working man down with typhoid fever. Think of the cost alone. Such a man needs to be seen at least once a day, and sometimes twice, by a doctor for a month; and after the month he should be visited two or three times a week for three weeks longer. He also needs nursing of the most intelligent kind. This is indispensable in every case of typhoid fever. Errors of management turn many slight cases into severe ones, and kill many patients who would otherwise recover. The food of a typhoid fever patient is most expensive, and must be so for several weeks.

Putting together the several items of medical attendance, proper nursing, food, physic, and loss of wages, the cost to a working man of a seven weeks' attack of typhoid fever cannot be less than from thirty to forty pounds. But this is ruin to a workman. It is a deadly blow to his prosperity, from which he can hardly ever recover. It may paralyse his efforts in the way of independence and self-help for years.

### The Poor Man's Pocket.

Now, think for a single moment what the hospital does for a poor man, even from this point of view. It saves him from every penny of money loss, except the deficiency in his earnings. It secures for him, in addition to competent medical aid, the two next most indispensable of all things in typhoid fever cases —intelligent nursing and proper food. In so doing the hospital not only greatly diminishes the sufferings resulting from typhoid fever, but it also shortens the attack and the convalescence, thus enabling a workman to return to his work at a much earlier date than he would under ordinary circumstances. Moreover, many lives of young and middle-aged men, suffering from typhoid fever, are annually saved by this intelligent management, that would be lost under less skilled guidance and control.

Taking this one serious and dangerous disease of typhoid fever alone, the general hospitals of London, excluding altogether the Poor Law Infirmaries, do not treat fewer than about 3,000 cases every year. Those 3,000 cases, as we have already shown, would entail a loss of from thirty to forty pounds each upon the workmen and their families, or not less than £10,000 altogether. The cost of these cases at the various hospitals is probably a good deal less than £10,000; but even if it be not, the outlay is charged entirely upon the more wealthy part of the community; that is to say upon those who give what they themselves consider they can really spare. The giving of the £10,000 is an advantage and a pleasure to those who bestow it; the enforced spending of it by the 3,000 typhoid fever workmen would be financial ruin instead of a pleasure to them.

### The Pains of Rheumatism.

Many readers know something about rheumatic fever, either in their own persons, or in the persons of their friends. Looked at from a patient's point of view, an attack of rheumatic fever is a calamity detestable, and almost intolerable. The mere pains it inflicts for days or weeks are in many cases the very utmost that a human being can endure, and continue to live. But what does rheumatic fever inflict upon a man, in addition to these agonising pains? In many cases it leaves him with such a disease of the heart as takes away at a single stroke fifteen or twenty years from the working value of his life. In addition to that, it makes him far more susceptible to future attacks than he ever was before. Moreover, rheumatic fever is often an illness which lasts longer than even typhoid. Its duration, including the period of convalescence, is from six weeks to six months. The London hospitals treat not fewer than from 5,000 to 6,000 cases of this disease annually. What could those five or six thousand poor men and women do in their miserable little homes under such circumstances as these? They would die by hundreds; and they would be permanently disabled almost by thousands. It is worth while spending fifty or sixty thousand a year in London for the treatment of rheumatic fever patients alone.

### Pneumonia: Our Charming Climate!

One more illustration of the serious classes of medical cases dealt with at the London hospitals is all we have space for, though it would be easy to fill a large volume without exhausting the facts or the interest of the subject. Most of us in London know something about pneumonia, which is popularly called inflammation of the lungs. Here is one fact which will strike the dullest imagination: If a hundred persons above fifty years of age were attacked with severe inflammation of the lungs not less than eighty of them would almost certainly die. It is startling to be told that at the London hospitals not fewer than from 9,000 to 10,000 severe chest cases, excluding consumption altogether, are treated every year, and that most of these are either cases of pneumonia or of acute bronchitis, which latter, in many patients is almost as dangerous as pneumonia. Now the least instructed reader understands that for cases of inflammation of the lungs and bronchitis, the first and most indispensable of all requisites is good air and plenty of it. A limited quantity of oxygen, and the presence of atmospheric impurities in the air breathed by patients of this class is exactly the same as administering deadly poison to them. But what kind of air is it that even the very best of the homes inhabited by London workmen can supply? Those homes are situated in the most crowded neighbourhoods; and the rooms into which each house is divided are little better than boxes. To treat a case of severe pneumonia in a house of this kind is almost infallibly to ensure the patient's death. Besides, let anybody consider the kind of nursing which such cases require. The nursing of a patient of this class is almost the chief part of the battle. A doctor treating cases of pneumonia without skilled nurses is like a general fighting a battle without an army. But what poor workman, whose wages are stopped by his illness, can afford to pay thirty shillings a week for a trained nurse? He might just as well be asked to pay thirty pounds, or three hundred. There is here no appeal made to sympathy. The hardest of hard facts are stated in the briefest possible way compatibly with their being intelligently comprehended by the reader.

### A Plethora of Facts.

Having entered upon this plain statement of facts, and being charged with an overwhelming quantity of new information obtained from the hospitals for the purposes of this article, the writer feels reluctant to give such a very small peep of what to many is an unknown country, when he is conscious that he has the means of exposing such a noble and expansive sight to view. But considerations of space are imperative. We cannot, however, close this paper without referring to two other classes of facts which bear upon the question of hospitals.

A few pages back reference was made to an accident which happened last Lord Mayor's Day, which came under the writer's personal cognisance. Now, if we were speaking to an audience instead of writing for readers we should pull our hearers up short by asking them, before stating the fact, to try to guess how many accidents are treated by the London hospitals during a single year? It is pretty certain that not one person in a hundred would guess anything like the real number. The writer himself, notwithstanding his twenty years' experience of hospital life, was startled by the actual facts. Our returns are not quite complete, and, therefore, we cannot give an exact statement for any particular recent year; but, making a computation from the returns actually to hand, we are justified in stating that there are probably between 250,000 and 300,000 accident cases treated by the metropolitan and suburban hospitals annually. Last year Guy's alone treated the enormous total of 20,766. Of these, 1,297 were accidents of a serious nature, such as broken legs, fractured thighs, smashed ribs, fractured skulls, dangerous injuries from falls or from falling buildings, and, indeed, every conceivable variety of hurt which can come to an industrial population that carries on its daily work in the hurrying streets of the largest and most crowded city in the world.

But, let the reader picture to himself 250,000 persons wounded and injured by accidental circumstances in fifty-two weeks! What sort of life campaign is this which kills and wounds so many, not only in a single year, but year by year, and more and more as our city expands, and our trade increases? Nearly five thousand persons every week taken to the hospital with injuries that are inflicted upon them in the course of their daily work. Almost a thousand a day for every working day! What can you possibly do with all these people if you have not free hospitals, with doors opened wide day and night in every part of London? Shut the doors of the hospitals! You may as well talk of cutting off the supply of the people's food! To a practical and responsible mind the hospitals are just as indispensable as daily clothing or daily bread.

### Bread Winners.

One of the questions addressed to the various hospital officials for the purposes of the present paper was the following: "What proportion of bread-winners do you ordinarily find among your in-patients?" That is, of course, among the really serious cases that seek for hospital treatment. The replies in all cases are very striking. More than eighty per cent. of all the patients treated for serious diseases are men and women engaged in the earning of their own bread and of the bread of those who are dependent upon them. Taking Guy's as a typical hospital, we find that the in-patients consist of at least eighty-three per cent. of wage-earners and bread-winners, the remaining 17 per cent. being children and young people. The proportion at the London Hospital is about the same. In other words, of the 9,500 serious cases that are treated within the wards of the London during the year, 7,300 not only belong to the wage-earning classes, but are themselves actually wage-earners. We have, therefore, every year in all the metropolitan hospitals an army of wage-earners in addition to all the other in-patients, and excluding the out-patients altogether, of not less than 100,000 persons suffering from serious, and, in many cases, prolonged and dangerous illness. If London were without hospitals, those honest and industrious persons would be absolutely destitute when attacked by fever or other sickness, and would die, as the cattle die of plague, by thousands and tens of thousands. The consequence to the community, and particularly to the ratepayers, of their illness and death, without hospital aid, would be incalculable, and, indeed, inconceivable.

### Only the Fringe of the Subject Touched.

The best of causes may suffer from incompetent advocacy. The writer has done his utmost to present a sample of the facts which constitute the daily work of hospitals, with the view of making that work speak for itself. He is conscious that his presentment of the case is altogether inadequate. He has left out of his narrative a thousand things which he might have put in; and some of those excluded facts bear the most eloquent testimony to the work which is daily being done. For example, the London Hospital informs us that it performs no less than 1000 serious surgical operations in the operation theatre in each year, besides the innumerable smaller operations that are performed in the wards and in the out-patient room. It treats 696 broken limbs, 420 burns and scalds, 491 serious wounds other than accidents, and 353 concussions of the brain. If these figures be multiplied by twelve they will still understate the work of all the medical charities of London.

### No Hospitals? Impossible!

How, then, can any man contemplate the possibility of a civilized London without hospitals? The Christian must support hospitals because his faith bids him do so, and the Jew for similar reasons. The economical man of business must support them because they save lives which are indispensable to the success of business, and shorten illnesses which, if lengthened, would diminish the profits of business. The ordinary man of the world must support them because it pains him more to know that a fellow creature is suffering without aid than it does to deny himself an occasional luxury in order to give aid to the suffering; the good woman must support them because her gentle and loving spirit flies to the relief of distress all the world over on an uncalculating divine impulse of the purest tenderness.

Civilization is impossible without hospitals, exactly as hospitals are impossible without civilization. Civilization carries with it literature, art, music, religion, mutual help, and that noblest and most indispensable of all the forms of charity—care and help for the sick. What more shall a man say who feels that British hospitals and the British Empire are bound together in indissoluble bonds? The clergyman, who has an intelligent appreciation of his duty, labours with all possible zeal and ardour for the elevation of human spirits and for the progress of his church; and is not that physician a traitor to his calling who does not magnify his office, and strive to bring the unspeakable blessings of his art to the aid of every human creature who is in sickness and distress? The cold cynicism which would pass by any suffering man, woman, or child on the plea of leaving them to the operation of Nature's laws, can only live in the hearts of men who would be murderers if murder were safe and convenient. The advancement of civilization and the progress of the human race are only possible on lines that run parallel with such institutions as hospitals and every other form of mutual relief and help. London without hospitals would be London without the spirit that creates hospitals; and London without the spirit that creates hospitals would be London doomed.

### The Plea of the Figures.

Hamlet stirred up his flagging spirit for the deed that awaited his hand by arguments that appealed to the nobler and manlier side of his nature:

> What is a man

> If his chief good and market of his time

> Be but to sleep and feed? A beast, no more,

> . . . . . I do not know,

> Why yet I live to say "this thing's to do."

> Sith I have cause and will and strength and means

> To do't.

> Examples gross as earth exhort me.

The writer labours to stir up all right-thinking men by the moving, but never misleading, plea of facts and figures. Although the amount of work done by the London hospitals during the year is the work of a kingdom, and is worthy of the revenues of a kingdom, they spend only the modest total of £606,258. But the public does not provide this sum in subscriptions every year; very little more, indeed, than half of it. The total amount subscribed by the population of London and of the surrounding districts last year was £320,662, a little more than a shilling for every head of the population. The invested funds of the institutions yielded £151,229. But we of the present generation must take no credit for this; it is the fruit of the good work of our fathers, our grandfathers, and our great grandfathers. An additional £50,194 was obtained from patients' payments; a very considerable sum, and one which will surprise a good many.

Putting these three items together, we only make a total income from all sources of £522,085. But we have just shown that the expenditure of the hospitals last year was £606,258. There was thus left as the result of the year's working the enormous deficit of £84,173. That deficit the Hospital Sunday Fund, as we all know, only succeeded in diminishing by a little more than half. What was the inevitable result? The hospitals had to sell out their invested capital to the tune of £30,000 or more. That was killing the goose that laid the golden eggs with a vengence.

The writer is informed on the most unquestionable authority that "the present year has been the very worst for procuring money for hospitals in the experience of the oldest living official." It is anticipated that the deficit on the operations of 1891, instead of being £84,000 as in 1890, will be not less than £150,000. These are stern figures for the poor and the suffering. It is not the hospital officials upon whom the indifference of so many of the public inflicts such untold injury. It is the industrious workman, "society's conscript," as Carlyle has called him; the workman, and his hardly tried wife, and his helpless children.

### A Reasonable and a Sympathising Press.

A reasonable and a sypathising press is the capable friend in whom the hospitals put their trust at the present moment. The public gives heed to the press. The hospitals, as representing the greatest of all the charitable works of the greatest empire in the world, appeal both to the press and the public. It is impossible to think without consternation of a deficit of £150,000 on the working of a single year. Such a state of things cannot be permitted to continue. However it is to be done, the hospitals must be delivered from the crutches and bandages of chronic poverty that compel them to lie down helplessly when they ought to walk, and to walk when they ought to run. The press, the daily and weekly press, can turn poverty into wealth if it will. Will it recognise a duty and an opportunity? Will it come to the help of the most human, and almost the most divine, of all the institutions of our England, and of the world?

---

According to a French contemporary peroxide of hydrogen has been found of great service in cases of diphtheria affecting the pharynx and nasal passages. The oxygenated water, which is powerfully antiseptic, should be diluted with about six times its bulk of pure water, and used as a gargle for the throat and a wash for the nose three times a day. In serious cases the treatment may be used every hour. Dr. Derlett's experience has led him to the conclusion that this treatment is useless if any general infection exists or the larynx is affected; but among its advantages may be enumerated the facts that no accidents need ensue, children bear the treatment well, false membranes are detached, and secretions are absorbed.

